# Grip strength is an important predictor for nutritional risk and early postoperative ambulation in gastrointestinal tumors undergoing laparoscopic surgery: a prospective multicenter clinical study

**DOI:** 10.1186/s12957-023-03163-x

**Published:** 2023-08-30

**Authors:** Jing Zhou, Xiao Liu, Xin Guo, Xiuxiu Yang, Xiaonan Ma, Weinan Liu

**Affiliations:** 1 Department of General Surgery, The First Medical Center, Chinese PLA General Hospital, Beijing, China; 2 The Sixth Medical Center, Chinese PLA General Hospital, Beijing, China; 3 Department of General Surgery, Peking Union Medical College Hospital, Chinese Academy of Medical Sciences, Beijing, 100730 China

**Keywords:** Gastrointestinal tumor, Early postoperative ambulation, Grip strength measurement, Nutritional risk

## Abstract

**Background:**

Using grip strength as a predictor of nutritional risk and early ambulation for gastrointestinal tumor surgery and determining its critical value have not been reported. This study was designed to explore the influencing factors of early postoperative ambulation ability for patients with gastrointestinal tumors who underwent laparoscopic surgery.

**Methods:**

Four-hundred twenty-seven patients with gastrointestinal tumors who underwent laparoscopic surgery at three tertiary A hospitals in Beijing were prospectively enrolled. Subsequently, logistic regression analysis was conducted to determine the independent predictors of early postoperative ambulation. Logistic regression analyses for the different gender were also performed. In addition, the effectiveness of preoperative grip strength measurement in nutritional risk assessment was analyzed by using nutritional risk score 2002 (NRS 2002) as a control.

**Results:**

The included cases were comprised of 283 male and 144 female patients, with an age of 59.35 ± 11.70 years. Gender, preoperative grip strength, operative time, and number of indwelling tubes were independent predictors of early postoperative ambulation. In the male group, lower preoperative grip strength and more indwelling tubes were independent risk factors for early postoperative ambulation. In the female group, lower preoperative grip strength and extended operating time were independent risk factors. Moreover, preoperative grip strength (male < 32 kg, female < 21 kg) can be used as a risk predictor for both preoperative nutritional risk and early postoperative ambulation.

**Conclusions:**

As a simple and objective measure of muscle strength, grip strength measurement is expected to be an effective predictor for both early postoperative ambulation ability and nutritional status of patients.

## Introduction

Enhanced recovery after surgery (ERAS) programs, which aims to reduce early complications, promote postoperative recovery, and shorten hospital stay by optimizing perioperative treatment and nursing measures, were first proposed and applied in clinic by Dr. Kehlet in the 1990s [1]. In recent years, this treatment model has been widely popularized and applied in China. As the common class IV operation of general and abdominal surgery, gastrointestinal tumor operation was the earliest and most widespread surgical field in which the ERAS protocol was applied [2, 3]. The optimization of postoperative analgesia, early ambulation, and rapid recovery of digestive tract function are considered as the key technologies of the ERAS model, among which early ambulation is an important strategy with easy operation, high feasibility, and strong safety in nursing work. According to previous studies, early ambulation after abdominal surgery could not only reduce venous thrombosis, pneumonia, and other complications but also promote digestive tract function recovery, wound healing, and sleep improvement [3–5].

In nursing practice, however, the implementation of early ambulation after abdominal surgery was not ideal. Many factors may contribute to this phenomenon [6, 7]. First, the definition of early ambulation was ambiguous, mainly reflected in the inconsistent start time and activity level. Second, medical personnel did not fully realize the importance of early ambulation, leading to the weak responsibility of assisting patients in early ambulation. Third, the pain and discomfort after major abdominal surgery and the traditional concept of bed rest lead to the poor compliance of patients and their families. Therefore, formulating appropriate standards and determining predictive indicators may effectively improve the implementation rate of early ambulation and reduce the occurrence of accidents such as falls.

Grip strength refers to the force generated by the combined contraction of the internal muscle group of the hand and the lateral muscle group of the forearm when gripping a target object with the hand under certain conditions. Grip strength measurement has the advantages of simple, convenient, rapid, objective, and non-invasive, which can be widely used in clinic. In previous studies, grip strength was associated with muscle strength, physical and cognitive function, and it predicted length of hospital stay and cost, as well as nutritional risk [8–10]. However, using grip strength as a predictor of early ambulation for gastrointestinal tumor surgery and determining its critical value have not been reported. The current study aimed to determine the relationship between preoperative grip strength and other predictors of early postoperative ambulation among patients undergoing gastrointestinal tumor surgery. Another aim was to determine whether preoperative grip strength was associated with preoperative nutritional risk.

## Materials and methods

### Study design and participants

Patients with gastrointestinal tumors who underwent laparoscopic radical surgery at three tertiary A hospitals (the First and Sixth Medical Center of Chinese PLA General Hospital and Peking Union Medical College Hospital) in Beijing from March 2022 to August 2022 were prospectively included in this study. A power analysis was conducted to determine the sample size. Patients with any of the following conditions were excluded from the study: (1) open surgery or conversion to open surgery, (2) non-gastrointestinal tumor surgery or combined with other organ surgery, (3) patients who transferred to intensive care unit (ICU) after surgery, (4) patients who did not get out of bed within 24 h after surgery, and (5) patients or family members refused to cooperate and participate. All procedures in this study were performed after obtaining informed consent from subjects or family members. This study was approved by the Medical Ethics Committee of the First Medical Center of the Chinese People’s Liberation Army (PLA) General Hospital (S2022-323–01).

### Variables collection

The following case data were prospectively collected: gender, age, body mass index (BMI), neoadjuvant chemotherapy, hypertension, diabetes, cerebrovascular disease, educational level, preoperative nutritional risk score 2002 (NRS 2002), operative time, intraoperative blood loss, application of analgesic pump, postoperative indwelling tube number, preoperative grip strength, serum hemoglobin (HGB), albumin (ALB), K + , and axillary temperature on the day of postoperative activity. Moreover, indwelling tubes include nasogastric tubes, drainage tubes, urinary tubes, and intravenous infusion tubes. Preoperative grip strength measurement, nutritional risk assessment, and related serum tests were completed on the day of admission or the next morning, and NRS 2002 score ≥ 3 was considered as nutritional risk.

### Outcome evaluation

Grip strength was measured using the standing measurement method specified in the Standard Manual of National Physical Fitness: Stand in a natural position with your arms at your sides, feet-shoulder width apart, and squeeze the gripometer as hard as you can with one hand. For critical patients who are unable to maintain a good standing or sitting posture, take a supine position with the head of the bed raised by 30°, keep arms straight and placed at the sides of the body, and then grasp the gripometer with the assistance of the nurse [11]. Subsequently, recording the reading of the gripometer to two decimal places, and alternating between the right and left hands twice for the average. The unit of grip strength was kg. The grip strength was measured at admission, on the day of surgery, and on each of the 3 days following surgery, for a total of five measurements. A new generation of electronic grip strength measuring instrument (CAMRY EH101) produced by Guangdong Senssun Weighing Instrument Group Ltd. was used, which had the characteristics of accurate measurement, reliable quality, simple operation, and convenient reading. At present, there is very little evidence to support the optimal time, intensity, and type of early ambulation, and unified evaluation methods, criteria, and implementation guidelines are lacking. Based on published literature, our clinical experience, and the completion of patients in this clinical trial, 30 m was set as the threshold of early ambulation distance. In this study, patients with an ambulation distance of ≥ 30 m within 24 h after returning to the ward were considered to accomplish early postoperative ambulation and were divided into the early ambulation group. Moreover, patients with ambulation distance < 30 m or start time > 24 h were considered to fail to accomplish early ambulation and were divided into the incomplete group. Early postoperative ambulation was completed with the assistance of family members or nursing staff.

The NRS 2002 score, first published in 2002, was the only nutritional risk screening tool that used “clinical outcome” as a criterion. In addition, it was recommended by the European Society for Parenteral Nutrition (ESPEN) and the Chinese Society for Parenteral Nutrition (CsPEN) as one of the nutritional risk screening tools for cancer patients and has been widely used. In this study, NRS 2002 score was used to evaluate the nutritional status of the subjects in hospital, including disease-related score, nutrition-related score, and age score. NRS 2002 score ≥ 3 was considered as nutritional risk, and NRS 2002 score < 3 was classified as no nutritional risk [12, 13].

### Statistical analysis

SPSS software (version 26.0) was used for the statistical analysis. The normal distribution of the continuous data was tested using a column diagram. If the data followed a normal distribution, the mean ± standard deviation (SD) was used to illustrate continuous variables and compared using the independent-samples *T*-test. Otherwise, the median (interquartile range, IQR) was calculated, and the Mann–Whitney *U*-test was applied in the inter-group comparison. Categorical data were depicted as numbers (percentages) and compared using the two-sided chi-squared or Fisher’s exact test. Variables with a *p*-value < 0.10 in the univariate analysis would be included in a multivariate binary logistic regression model, and variables with a *p*-value < 0.05 in the multivariate analysis were considered as independent predictors. In addition, with early postoperative ambulation and preoperative nutritional risk as state variables, the receiver operating characteristic (ROC) curves of preoperative grip strength were plotted to calculate the Yoden indexes, and the critical value corresponding to the maximum Yoden index was taken as the cut-off value.

## Results

### Study population characteristics

**Fig. 1:**
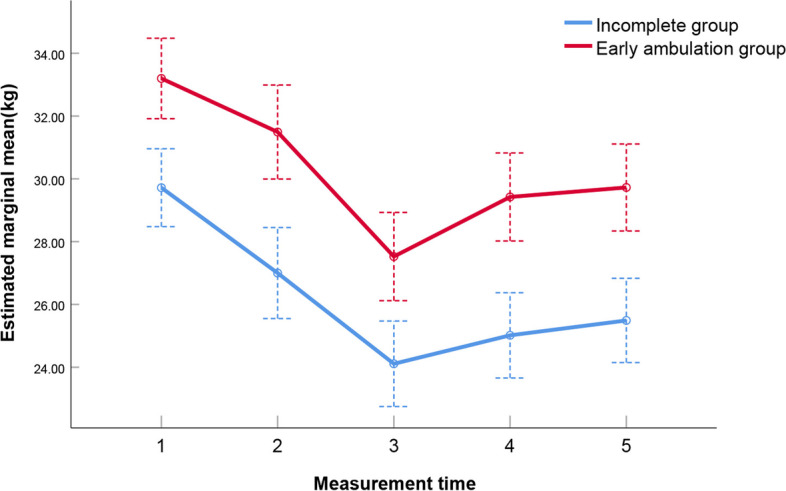
Comparison and variation tendency of grip strength between the early ambulation and incomplete group at each time point

After screening, a total of 427 patients who underwent laparoscopic gastrointestinal tumor surgery were enrolled, including 283 male and 144 female patients, with an age of 59.35 ± 11.70 years. Based on the completion of early ambulation, 427 patients were divided into early ambulation and incomplete group, including 274 and 153 patients, respectively. The grip strength of the two groups was measured at admission, on the day of surgery, and on each of the 3 days following surgery, for a total of five measurements. The values of the early ambulation group were 30.82 ± 8.20 kg, 28.81 ± 8.90 kg, 25.92 ± 8.72 kg, 27.02 ± 8.85 kg, and 27.45 ± 8.61 kg, while the grip strength measurements of the incomplete group were 24.80 ± 7.70 kg, 22.50 ± 8.93 kg, 19.01 ± 7.13 kg, 20.43 ± 7.20 kg, and 20.68 ± 7.48 kg, respectively. Further analyses showed statistical differences in grip strength between the two groups at each time point (*p* < 0.01), and the grip strength of both groups decreased first and then increased and reached the lowest on the first day after surgery (Fig. 1).

### Univariate analysis for early ambulation ability

Univariate analysis showed that the age (*p* = 0.047), educational level (*p* = 0.029), preoperative NRS 2002 score (*p* = 0.005), operative time (*p* = 0.001), application of analgesic pump (*p* = 0.006), postoperative indwelling tube number (*p* = 0.009), preoperative grip strength (*p* < 0.001), and serum ALB (*p* = 0.011) were associated with early ambulation. There were no statistically significant differences between early ambulation and incomplete group in gender, BMI, neoadjuvant chemotherapy, hypertension, diabetes, cerebrovascular disease, intraoperative blood loss, serum HGB, K + , and axillary temperature on the day of postoperative activity (Table 1).

**Table 1:** Characteristics of included patients in the early ambulation group and incomplete group

Variables	Total (*n* = 427)	Early ambulation group (*n* = 274)	Incomplete group (*n* = 153)	*p*-value
Gender				0.073
Male	283 (66.3)	190 (69.3)	93 (60.8)	
Female	144 (33.7)	84 (30.7)	60 (39.2)	
Age (years)	60 (52, 68)	59 (51, 68)	61 (55, 68)	**0.047**
BMI (kg/m^2^)	24.0 (22.1, 26.4)	24.0 (22.1, 26.5)	23.8 (21.8, 26.2)	0.416
Neoadjuvant chemotherapy				0.938
Yes	44 (10.3)	28 (10.2)	16 (10.5)	
No	383 (89.7)	246 (89.8)	137 (89.5)	
Hypertension				0.635
Yes	126 (29.5)	83 (30.3)	43 (28.1)	
No	301 (70.5)	191 (69.7)	110 (71.9)	
Diabetes				0.864
Yes	57 (13.3)	36 (13.1)	21 (13.7)	
No	370 (86.7)	238 (86.9)	132 (86.3)	
Cerebrovascular disease				0.477
Yes	26 (6.1)	15 (5.5)	11 (7.2)	
No	401 (93.9)	259 (94.5)	142 (92.8)	
Educational level				**0.029**
Primary	71 (16.6)	45 (16.4)	26 (17.0)	
Middle	102 (23.9)	54 (19.7)	48 (31.4)	
High	104 (24.4)	68 (24.8)	36 (23.5)	
University	150 (35.1)	107 (39.1)	43 (28.1)	
Preoperative NRS 2002 score				**0.005**
≥ 3	155 (36.3)	86 (31.4)	69 (45.1)	
< 3	272 (63.7)	188 (68.6)	84 (54.9)	
Operative time (h)	3.2 (2.3, 4.0)	3.1 (2.3, 3.8)	3.3 (2.6, 4.3)	**0.001**
Intraoperative blood loss (ml)	50 (30, 100)	50 (30, 100)	50 (30, 100)	0.593
Application of analgesic pump				**0.006**
Yes	328 (76.8)	222 (81.0)	106 (69.3)	
No	99 (23.2)	52 (19.0)	47 (30.7)	
Postoperative indwelling tube number	3 (3, 4)	3 (3, 4)	4 (3, 4)	**0.009**
Preoperative grip strength (kg)	28.4 (22.4, 35.2)	32.4 (24.6, 36.9)	24.5 (19.4, 29.1)	** < 0.001**
Serum HGB (g/day)				0.366
≥ 120 (male) or ≥ 110 (female)	326 (76.3)	213 (77.7)	113 (73.9)	
< 120 (male) or < 110(female)	101 (23.7)	61 (22.3)	40 (26.1)	
Serum ALB (g/day)				**0.011**
≥ 35	384 (89.9)	254 (92.7)	130 (85.0)	
< 35	43 (10.1)	20 (7.3)	23 (15.0)	
Serum K^+^ (mmol/L)				0.486
≥ 3.5	380 (89.0)	246 (89.8)	134 (87.6)	
< 3.5	47 (11.0)	28 (10.2)	19 (12.4)	
Axillary temperature on the day of postoperative activity				0.348
> 37	109 (25.5)	74 (27.0)	35 (22.9)	
≤ 37	318 (74.5)	200 (73.0)	118 (77.1)	

Data are reported as numbers (percentage), mean ± standard deviation, or the median (interquartile range, IQR)

*BMI* body mass index, *NRS* nutritional risk score, *HGB* hemoglobin, *ALB* albumin

### Multivariate analysis for early ambulation ability

The variables contained gender, age, educational level, preoperative NRS 2002 score, operative time, application of analgesic pump, postoperative indwelling tube number, preoperative grip strength, and serum ALB were further included in multivariate logistic regression analysis. Multivariate analysis revealed that the gender (odds ratio (OR) = 0.511, *p* = 0.034), preoperative grip strength (*OR* = 1.129, *p* < 0.001), operative time (*OR* = 0.767, *p* = 0.004), and postoperative indwelling tube number (*OR* = 0.744, *p* = 0.011) were independent predictive factors of early ambulation (Table 2).

**Table 2:** Multivariate analysis of early postoperative ambulation for patients with gastrointestinal tumors

Variables	Coefficients	OR (95% *CI*)	*p*-value
Gender	− 0.671	0.511 (0.275, 0.951)	**0.034**
Age (years)	0.005	1.005 (0.984, 1.028)	0.632
Educational level			0.197
Primary vs middle	− 0.682	0.506 (0.254, 1.009)	0.053
Primary vs high	− 0.187	0.829 (0.412, 1.670)	0.600
Primary vs university	− 0.179	0.836 (0.420, 1.663)	0.610
Preoperative grip strength (kg)	0.122	1.129 (1.082, 1.179)	** < 0.001**
Serum ALB < 35 g/L	0.030	1.030 (0.485, 2.186)	0.938
Preoperative NRS 2002 score ≥ 3	0.163	1.177 (0.679, 2.039)	0.562
Operative time (h)	− 0.265	0.767 (0.640, 0.919)	**0.004**
Postoperative indwelling tube number	− 0.295	0.744 (0.593, 0.933)	**0.011**
Application of analgesic pump	0.520	1.682 (0.941, 3.004)	0.079
Constant values	− 0.993	0.370	0.364

*ALB* albumin, *NRS* nutritional risk score, *OR* odds ratio; *CI*, credible interval

Owing to the significant influence of gender on grip strength, univariate and multivariate analyses were performed separately for male and female patients. The univariate analysis based on male patients showed that preoperative grip strength (*p* < 0.001), serum HGB (*p* = 0.038), ALB (*p* = 0.020), preoperative NRS 2002 score (*p* = 0.010), operative time (*p* = 0.009), application of analgesic pump (*p* = 0.013), and postoperative indwelling tube number (*p* = 0.008) were predictors for early ambulation (Table 3a). Further multivariate logistic regression analysis showed that the lower preoperative grip strength (*OR* = 1.109, *p* < 0.001) and more postoperative indwelling tubes (*OR* = 0.666, *p* = 0.006) were independent risk factors (Table 3b).

**Table 3:** Univariate and multivariate logistic analyses for early postoperative ambulation in patients with gastrointestinal tumors (male group)

Variables	Coefficients	OR (95% *CI*)	*p*-value
**a. Univariate analysis**			
Preoperative grip strength (kg)	0.115	1.121 (1.077, 1.167)	< 0.001
Serum HGB < 120 g/day (male) or < 110 g/day (female)	0.634	1.886 (1.036, 3.431)	0.038
Serum ALB < 35 g/L	0.966	2.629 (1.163, 5.940)	0.020
Preoperative NRS 2002 score ≥ 3	− 0.672	0.511 (0.306, 0.852)	0.010
Operative time (h)	− 0.255	0.775 (0.639, 0.938)	0.009
Postoperative indwelling tube number	− 0.336	0.714 (0.557, 0.917)	0.008
Application of analgesic pump	0.732	2.079 (1.170, 3.693)	0.013
**b. Multivariate analysis**			
Preoperative grip strength (kg)	0.103	1.109 (1.057, 1.163)	** < 0.001**
Serum HGB < 120 g/day (male) or < 110 g/day (female)	0.053	1.054 (0.506, 2.195)	0.888
Serum ALB < 35 g/L	0.101	1.106 (0.396, 3.091)	0.847
Preoperative NRS 2002 score ≥ 3	0.056	1.058 (0.564, 1.985)	0.861
Operative time (h)	− 0.141	0.869 (0.704, 1.072)	0.189
Postoperative indwelling tube number	− 0.407	0.666 (0.499, 0.889)	**0.006**
Application of analgesic pump	0.692	1.998 (0.958, 4.168)	0.065
Constant values	− 1.286	0.276	0.202

*HGB* hemoglobin, *ALB* albumin, *NRS* nutritional risk score, *OR* odds ratio, *CI* credible interval

**Fig. 2:**
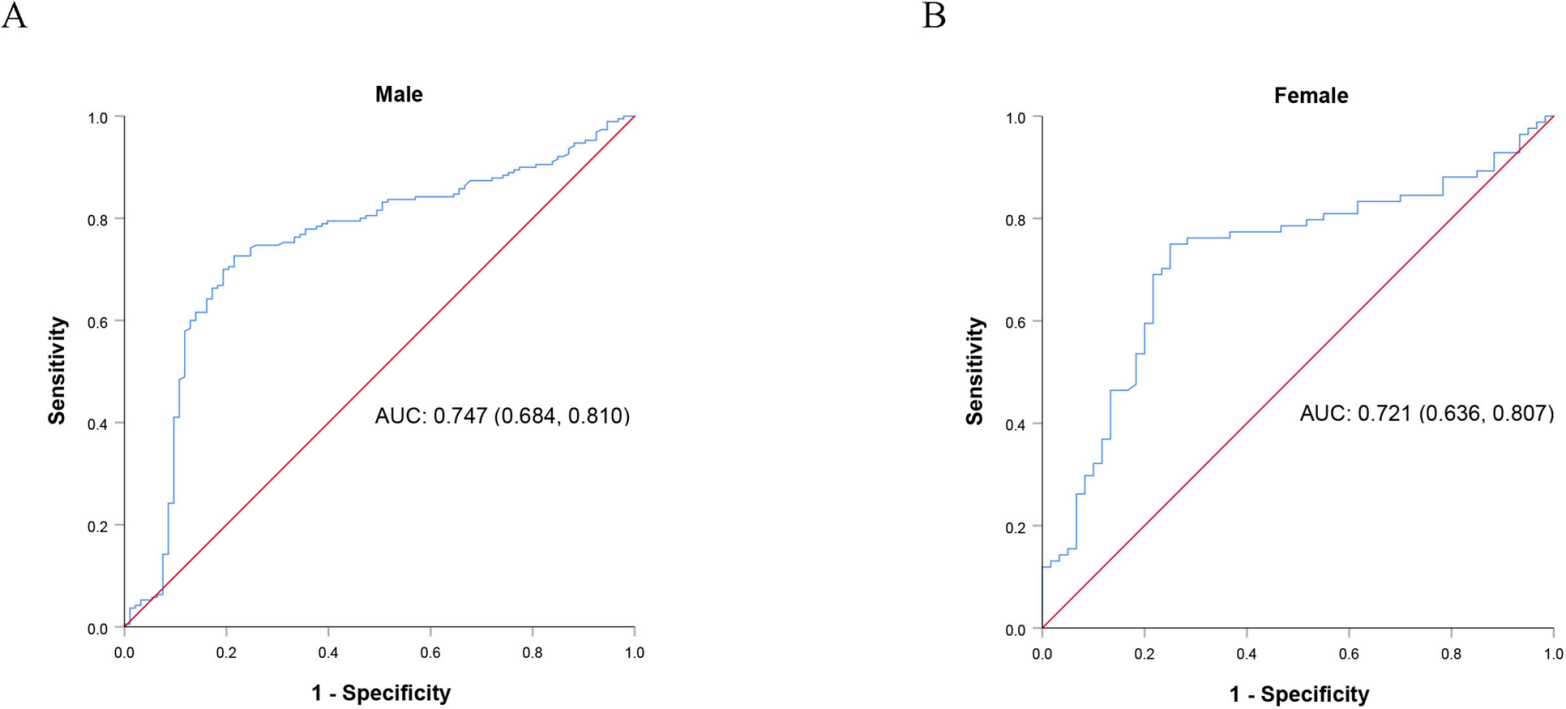
ROC curves of preoperative grip strength in **A** male and **B** female groups with the early ambulation condition as the state variable. ROC, receiver operating characteristic

Similarly, univariate analysis for female patients revealed that age (*OR* = 0.964, *p* = 0.011), preoperative grip strength (*OR* = 1.171, *p* < 0.001), and operative time (*OR* = 0.578, *p* < 0.001) were associated with early ambulation (Table 4a), while multivariate analysis identified the lower preoperative grip strength (*OR* = 1.148, *p* = 0.001) and prolonged operative time (*OR* = 0.587, *p* = 0.001) as independent risk factors (Table 4b). The ROC curves of preoperative grip strength for male (Fig. 2a) and female group (Fig. 2b) were plotted with the early ambulation as state variable, and the cut-off values were 31.9 kg and 20.7 kg, respectively.

**Table 4:** Univariate and multivariate logistic analyses for early postoperative ambulation in patients with gastrointestinal tumors (female group)

Variables	Coefficients	OR (95% *CI*)	*p*-value
**a. Univariate analysis**			
Age (years)	− 0.037	0.964 (0.937, 0.992)	0.011
Preoperative grip strength (kg)	0.158	1.171 (1.086, 1.263)	< 0.001
Operative time (h)	− 0.547	0.578 (0.429, 0.779)	< 0.001
**b. Multivariate analysis**			
Age (years)	− 0.009	0.991 (0.958, 1.026)	0.619
Preoperative grip strength (kg)	0.138	1.148 (1.056, 1.247)	**0.001**
Operative time (h)	− 0.532	0.587 (0.423, 0.815)	**0.001**
Constant values	− 0.371	0.690	0.829

*OR* odds ratio, *CI* credible interval

### Effect analysis of preoperative grip strength in nutritional risk assessment

**Fig. 3:**
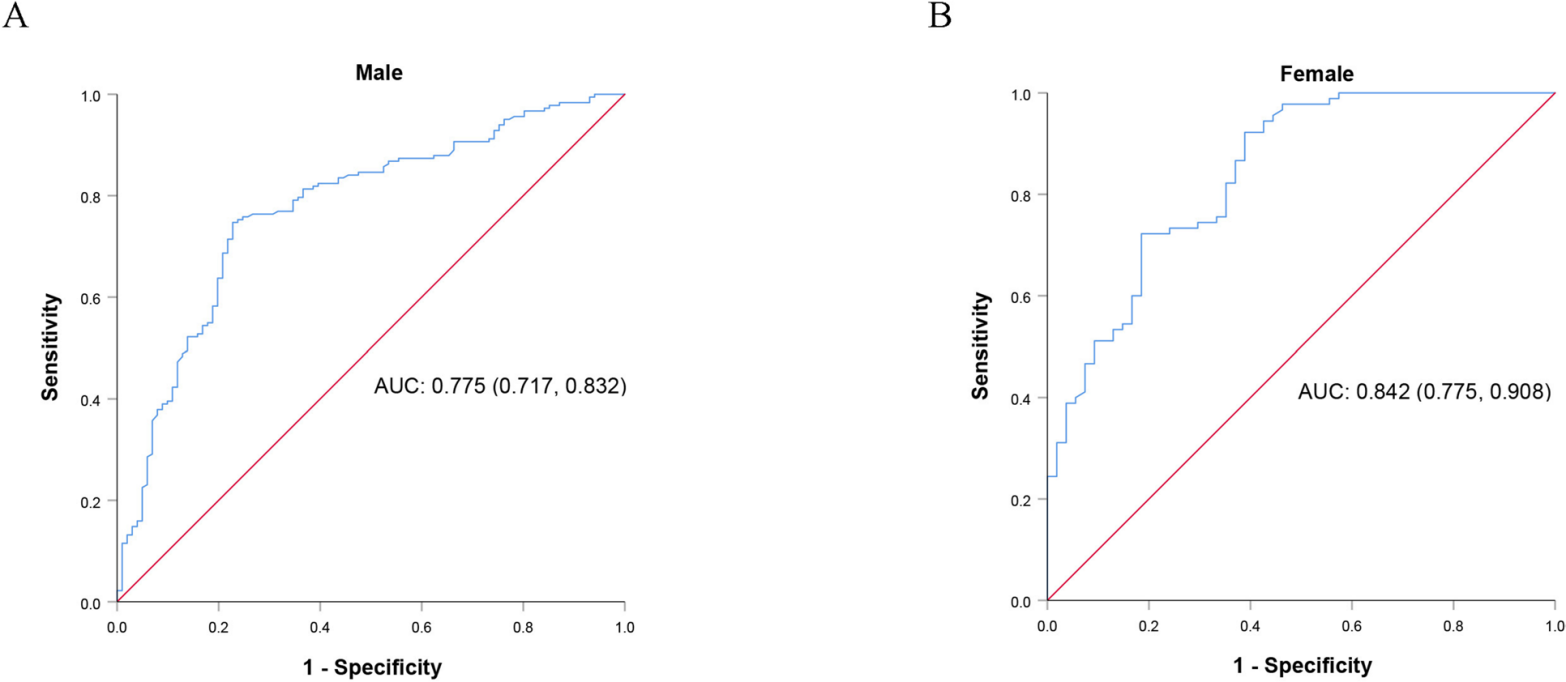
ROC curves of preoperative grip strength in **A** male and **B** female groups with the NRS 2002 score as the state variable. ROC, receiver operating characteristic

According to the assessment results of NRS 2002 at admission, the enrolled patients were divided into the nutritional risk (NRS 2002 ≥ 3) and non-nutritional risk group (NRS 2002 < 3). The ROC curves of preoperative grip strength for male (Fig. 3a) and female group (Fig. 3b) were plotted with the nutritional risk as state variable, and the cut-off values were 31.9 kg and 21.0 kg, respectively.

## Discussion

In this study, we quantified the patients’ early ambulation according to the start time and distance of postoperative activities [14]. As analysis results showed, the higher preoperative grip strength, less indwelling tubes, and shortened operative duration contributed to early postoperative ambulation. To avoid the influence of dominant hand on grip strength, in this study, the average value of bilateral grip strength was calculated, and repeated measurements were made to further improve the accuracy of results. Gender is known to be one of the main factors affecting grip strength [9], and our analysis also showed that there was a significant difference in the sex ratio between the early ambulation and incomplete group. Therefore, we grouped patients by gender and analyzed them separately. It was found that male patients with preoperative grip strength ≥ 31.9 kg and female patients with grip strength ≥ 20.7 kg were more likely to accomplish early postoperative activities.

The five grip strength values of early ambulation group were significantly higher than those of incomplete group, and the grip strength of two groups both decreased first and then increased and reached the lowest on the first day after surgery. Preoperative intestinal preparation, fasting on the day of surgery, and the patients’ tension and anxiety before surgery could result in a significant decrease of body state and muscle strength on the day of surgery compared with admission. Anesthesia, surgical bleeding, fluid loss and energy expenditure, trauma, pain, and postoperative fasting could lead to a further decline of muscle strength on the first day after surgery.

With the relief of postoperative pain, nutritional supplement, and state recovery, the muscle strength of patients gradually returned to the normal state before surgery from the second day after surgery.

Grip strength is an important reflection of the overall condition of skeletal and muscular systems, which are the body’s main stores of protein [15]. Malignant tumor is a wasting disease, during which metabolic changes such as decreased activity of mitochondrial complex, decreased synthesis of phosphocreatine, and increased intracellular calcium flow can occur. Moreover, increased protein decomposition and decreased protein synthesis are caused by tumor consumption, leading to the decreased skeletal muscle function and muscle atrophy; thus, the patient’s overall muscular strength, nutritional status, and grip strength are significantly decreased [16–18]. Previous studies have shown that the declines in muscular function precede declines in muscular mass. Grip strength not only is a sensitive indicator of short-term changes in the function of whole muscle groups but also reflects the storage of muscle protein to some extent [18]. Therefore, grip strength measurement can be used to predict the changes in nutritional status and muscle strength of patients as early as possible, so as to carry out nutritional intervention and functional exercise.

The number of indentured tubes was another important factor affecting patients’ early postoperative ambulation. First, because gastrointestinal tumor surgery involves local resection and reconstruction of digestive tract, there are more strict requirements on fasting time. During fasting, only peripheral vein, central vein, or nasal jejunal nutrient tube can be used for liquid and nutrition solution input, resulting in long postoperative infusion time and many lines [19]. Second, although laparoscopic surgery has less trauma and postoperative pain, in order to timely discover possible postoperative complications such as digestive tract fistula and bleeding, as well as to meet the needs of hyperthermic intraperitoneal chemotherapy (HIPEC), some patients have more abdominal drainage tubes [20]. Third, the long operating time for gastrointestinal tumors leads to the indwelling of catheters. The existence of the above various pipelines causes inconvenience and anxiety in the early postoperative activities, so most patients are reluctant to get out of bed. Additionally, the pain and discomfort caused by tube moves during physical activity also hinder the early postoperative activity [4, 21, 22]. As this analysis revealed, more postoperative catheter indwelling was not conducive to early ambulation. Thus, except for necessary infusion and drainage, the placement of additional infusion and drainage pipes should be avoided. The urinary and gastric tube should be removed early after the operation, which is convenient to get out of bed and also conducive to the recovery of gastrointestinal and bladder functions [21, 23, 24].

The analysis, based on the data from 427 patients, showed that the operative time was significantly shorter in the early ambulation group. The reasons for the influence of operative time on postoperative mobility are complicated. Prolonged operative time is often accompanied by difficult surgery, more intraoperative bleeding, and greater trauma, resulting in more intense postoperative discomfort and pain. Moreover, difficult operations often require more abdominal drainage indenture, not easy to carry out early postoperative activities. Longer anesthesia time and more intraoperative fluid intake, coupled with the protein loss caused by intraoperative bleeding, exudation, and edema, lead to poor postoperative nutritional status and listlessness of patients, which is also not conducive to early postoperative activities [24–26].

Although it has been reported that grip strength was closely related to the nutritional status of patients, few studies have taken grip strength as an indicator of preoperative nutritional risk assessment for malignant tumors [16–18, 27, 28]. Lu’s study showed that grip strength was an important predictor of nutritional risk for patients with malignant tumors, and its ROC curve area for predicting nutritional risk was > 0.7 [9]. Lu’s study, however, included malignancies of multiple systems, reducing the applicability and accuracy of the analysis results. In this study, we used the evaluation results of NRS 2002 as the control, and analyzed the correlation between preoperative grip strength and nutritional status for gastrointestinal tumors, and then found that preoperative grip strength could be used as a predictor of nutritional status. Furthermore, the cut-off values used to distinguish nutritional risk were extremely close to those used to assess early postoperative activity, suggesting that the cut-off values of preoperative grip strength obtained in this analysis (32 kg for male and 21 kg for female) could be used to predict both preoperative nutritional risk and early postoperative activity. But the predictive effect of this analysis needs to be validated with more patients’ data.

While this study enrolled numerous patients from three tertiary A hospitals and collected as much variable data as possible, there still remain several limitations. First, the three hospitals involved in this study were all from the same part of the same country, making wider generalization difficult. Randomized controlled studies involving multiple races from around the world would be more ideal, but it was not feasible since we cannot have access to the nurses in other countries and territories. Second, since there was no uniform definition and implementation guidelines for early ambulation, in this study, we quantified the early postoperative ambulation based on the initial time and activity intensity, and established the eligibility criteria of early ambulation, which needs to be further evaluated and validated. Third, the influence of activity duration was not considered in the early ambulation assessment, and the grip strength measurement and early ambulation assessment for multiple centers were not carried out by unified personnel, reducing the accuracy and persuasiveness of outcome indicators to some extent. Some patients with complications from surgery were removed from this analysis, limiting the generalizability of this study. Consequently, in the future, unified standards and guidelines for early postoperative ambulation are needed, and large-sample prospective researches involving medical centers from multiple countries and regions should be conducted to further validate and supplement the results of this analysis.

## Conclusion

As this analysis based on 427 patients’ data showed, preoperative grip strength, operative time, and postoperative indwelling tube number were independent predictors of early postoperative ambulation. For male patients, lower preoperative grip strength and more postoperative indwelling tube were independent risk factors for early ambulation. And for female patients, lower preoperative grip strength and prolonged operative time were proved to be independent risk predictors. In addition, preoperative grip strength < 32 kg (male) and < 21 kg (female) was risk predictors of both preoperative nutritional risk and early postoperative activity. As an indispensable part of ERAS program, early postoperative activity is an important measure for nurses to integrate ERAS concept into clinical nursing work. Moreover, as a simple and easy method to measure muscle strength, grip strength measurement is expected to be an effective predictor of early postoperative ambulation and nutritional status.

## Data Availability

The datasets used and/or analyzed during the current study are available from the corresponding author on reasonable request.

## Abbreviations

NRS: Nutritional risk score
ERAS: Enhanced recovery after surgery
ICU: Intensive care unit
PLA: People’s Liberation Army
BMI: Body mass index
HGB: Hemoglobin
ALB: Albumin
SD: Standard deviation
IQR: Interquartile range
ROC: Receiver operating characteristic
OR: Odds ratio
HIPEC: Hyperthermic intraperitoneal chemotherapy
